# Optimization of Distal Radial Access Into Clinical Routine Practice Through Ultrasound Guidance: A Review and Technical Guide

**DOI:** 10.31083/RCM46471

**Published:** 2026-02-26

**Authors:** Claudiu Ungureanu, Giuseppe Colletti, Steven Haine, Gregory Angelo Sgueglia

**Affiliations:** ^1^Cardiology Department, Jolimont Hospital, 7100 La Louvière, Belgium; ^2^Cardiology Department, Clinique Saint-Joseph, Groupe Vivalia, 6700 Arlon, Belgium; ^3^Department of Cardiology, Universitair Ziekenhuis Antwerpen (UZA), University of Antwerp (UA), 2000 Antwerp, Belgium; ^4^Division of Cardiology, Sant’Eugenio Hospital, 00144 Rome, Italy

**Keywords:** cardiac catheterization, distal radial artery, interventional cardiology, vascular access, ultrasound

## Abstract

Distal radial access (DRA) has emerged as a preferred approach in cardiac and peripheral vascular catheterization, offering distinct advantages over traditional access methods. However, DRA is inherently more challenging due to the smaller diameter, sharper angulation, and greater anatomical variability of the vessels, which collectively increase the risk of puncture failure and the need for crossover to alternative vascular access. Thus, ultrasound guidance has become increasingly important. Unlike the conventional transradial approach, where ultrasound guidance remains optional, the use of ultrasound guidance in DRA could offer additional benefits, including potentially improved success rates and a reduced risk of damage to surrounding anatomical structures. This review highlights the essential role of vascular ultrasound in DRA and presents a detailed, step-by-step guide that integrates sonographic and anatomical techniques. Therefore, by promoting technical precision and ensuring safer vascular access, this approach aims to optimize the success and safety of catheterization procedures and foster the widespread adoption of DRA in clinical practice.

## 1. Introduction 

Recently, distal radial access (DRA), particularly in the anatomical snuffbox, has gained significant interest for cardiac catheterization as evidence accumulates of its procedural and clinical benefits over other access sites [1, 2, 3]. This vascular access offers advantages such as a reduced risk of radial artery occlusion (RAO), faster post-procedural hemostasis, and improved patient comfort [2]. Another key advantage of DRA lies in its suitability for the left radial approach. The ergonomic comfort of left DRA for both patient and operator: with the palm facedown the patient can comfortably place the left hand over the right groin, reproducing a setup that closely resembles right femoral access. This approach provides an ideal coaxial alignment for engaging the left coronary system, facilitates catheter manipulation with greater stability, and represents an excellent alternative to femoral access, particularly for complex coronary interventions [4].

Despite its more distal location, DRA has been shown to accommodate large-bore guiding catheters (7–8 Fr) in carefully selected patients. This expands its potential use beyond diagnostic and standard PCI to include complex coronary procedures and even structural interventions requiring large-caliber devices [5].

However, accessing the distal radial artery presents certain challenges, which are reflected by the relatively higher rate of crossover to alternative vascular access sites [3]. This is typically due to the difficulty and longer time needed to puncture and cannulate the artery successfully. These challenges can hinder the potential diffusion of DRA and compel operators to switch to more familiar access routes.

The use of ultrasound (US) guidance in DRA substantially enhances the procedure’s success rate [6, 7]. Indeed, US guidance provides real-time visualization that enables precise identification and localization of the distal radial artery, facilitating its differentiation from surrounding structures such as veins, tendons, and nerves [8]. This enhanced precision has the potential to improve the likelihood of achieving successful puncture on the first attempt, thereby minimizing the number of attempts required. Consequently, US guidance may shorten access times and decrease the need for crossover to alternative access sites. Additionally, it could lower the risk of complications, such as hematoma formation and damage to adjacent tendons or nerves [8]. Beyond improving overall procedural success, ultrasound guidance may also help overcome specific anatomical challenges. Recent findings have highlighted that women may experience a higher rate of minor vascular access-site bleeding events with DRA, despite the smaller vessel caliber [9]. While this finding may be partly attributable to the very low body size observed in some of these patients, it also highlights the crucial role of ultrasound guidance, which allows direct evaluation of vessel size and course, optimization of the puncture angle, and selection of the most appropriate entry point.

This review underscores the critical role of US guidance in the routine practice of DRA and provides a detailed, step-by-step framework for its effective implementation. By improving puncture precision and procedural safety, US-guided DRA can enhance clinical outcomes and support the broader adoption of this access technique in contemporary practice.

## 2. Anatomic Considerations

### 2.1 Distal Course of the Radial Artery 

A thorough understanding of the anatomy of the distal radial artery is essential to avoid complications, including damage to the superficial branch of the radial nerve, arteriovenous fistula formation, and radial artery aneurysms that can result from repeated puncture attempts [7]. The radial artery runs along the outer side of the forearm, just above the radius bone, and continues toward the wrist. As it approaches the distal end of the radius, before crossing into the anatomical snuffbox, the artery gives rise to the superficial palmar branch.

This branch runs medially, passing through or near the thenar muscles, often remaining close to the skin. Its path allows it to join with the terminal part of the ulnar artery, contributing to the formation of the superficial palmar arch [10]. This arch plays a key role in providing adequate blood flow to the fingers and palm, which is essential for both sensation and movement.

**Fig. 1.**
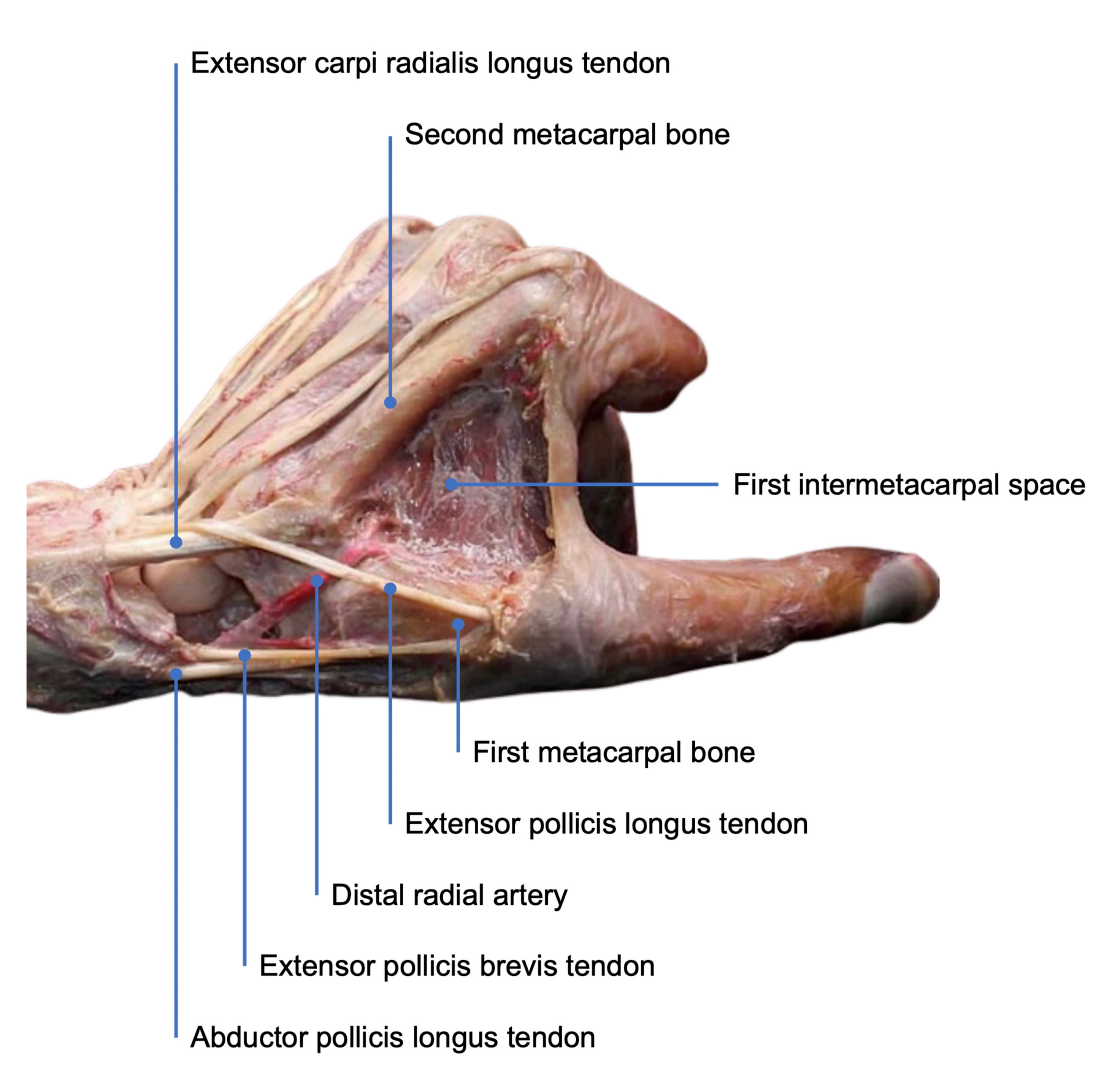
**Anatomical snuffbox structures**. Detailed anatomical dissection highlighting the key structures that form the anatomical snuffbox.

Further, along its course, the radial artery enters the anatomical snuffbox deep to the abductor pollicis longus and extensor pollicis brevis tendons. In most cases, the radial artery traverses the anatomical snuffbox near its base rather than more distally (Fig. 1) [11]. The anatomical variations in the curvilinear course of the distal radial artery and its relationship to surrounding structures make catheterization more challenging than conventional access locations. The vessel crosses the anatomical snuffbox somewhat obliquely and travels deep to the extensor pollicis longus tendon to traverse the first dorsal interosseous muscle [10]. Beyond the anatomical snuffbox, the artery continues superficially across the first intermetacarpal space on the dorsum of the hand, at the junction of the extensor pollicis longus tendon and the second metacarpal bone. After a very short superficial tract under the skin, where the pulse is much easier to detect, the trajectory of the artery becomes deeper, making it much more difficult to compress, especially in the absence of a firm bony floor. Finally, the radial artery curves medially between the heads of the first dorsal interosseous muscle and enters the palm, where it may connect with the deep branch of the ulnar artery to form the deep palmar arch [12].

While our understanding of distal radial artery anatomy has traditionally been based on cadaveric studies, these may not fully capture the dynamic nature of *in vivo* anatomy or account for individual variations and postmortem changes, particularly given the artery’s course through loose subcutaneous tissue.

### 2.2 Anatomical Snuffbox

The anatomical snuffbox is a triangular area on the back of the hand, bounded laterally by the tendons of the abductor pollicis longus and extensor pollicis brevis muscles, and medially by the tendon of the extensor pollicis longus (Fig. 1). This shallow, well-defined region is suitable for vascular puncture due to its accessible location. Yet, it contains other important structures, including the cephalic vein and a branch of the radial nerve, which must be carefully navigated when implementing DRA [10].

The cephalic vein is most commonly found in the more medial part and the superficial branch of the radial nerve divides into three terminal branches—lateral, intermediate, and medial—highlighting the complexity of the structures within the anatomical snuffbox and the need for precise anatomical knowledge during medical interventions.

The floor of the snuffbox consists of the distal radius, scaphoid, trapezium, and the base of the first metacarpal bone. Historically, this anatomical region was used by some to hold small amounts of tobacco (snuff) for inhalation.

## 3. Evidence for Ultrasound Guidance in Radial Access: From Conventional to Distal Approach

**Fig. 2.**
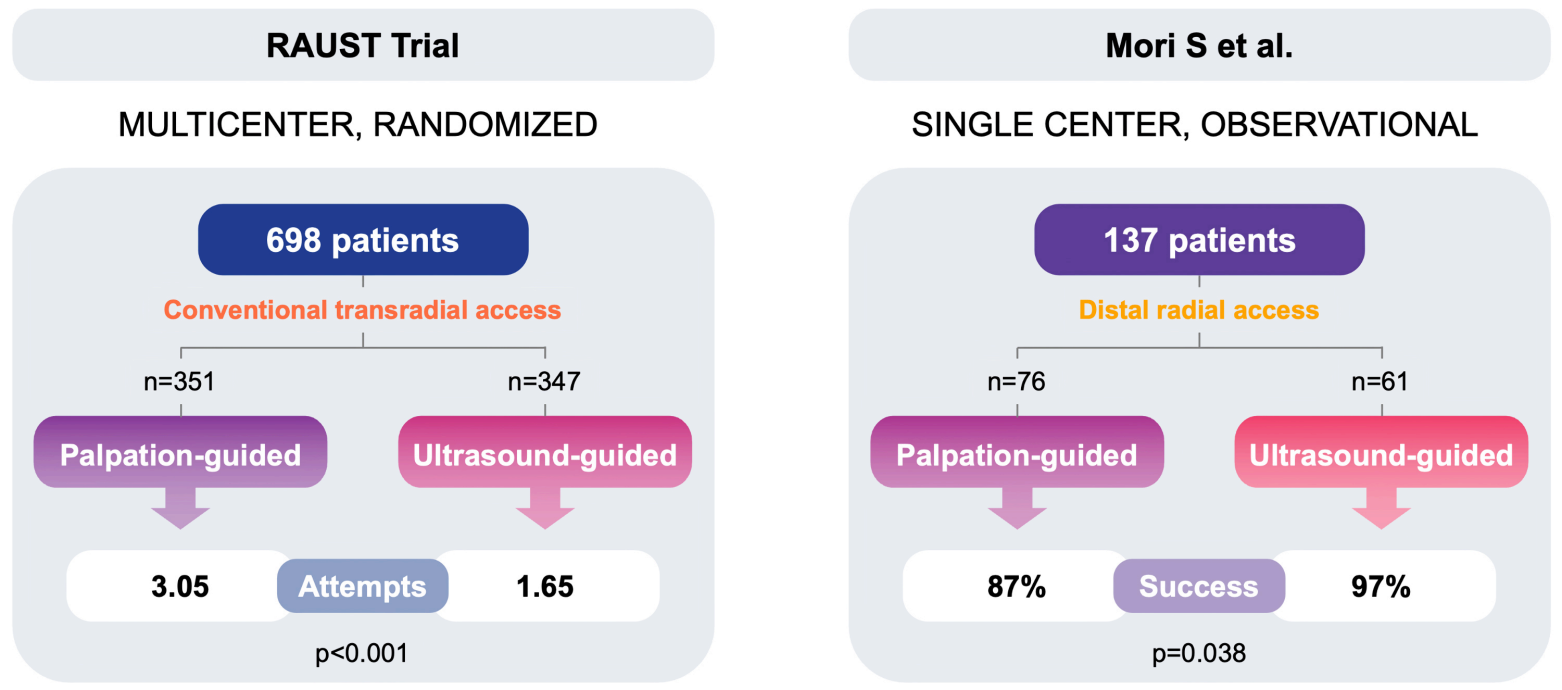
**Results of ultrasound-guided radial access**. The left panel outlines the key features of ultrasound guidance for conventional transradial access, while the right panel highlights the key aspects of ultrasound guidance for distal radial access. This figure was created using data reported in [13, 15].

In the Radial Artery Access With Ultrasound Trial (RAUST) randomized controlled trial (RCT), 698 patients undergoing conventional transradial access coronary procedures were randomized to needle insertion with either palpation or real-time US guidance [13]. In this trial, the number of attempts was lower in the US guidance group (1.65 ± 1.2 vs. 3.05 ± 3.4, *p* < 0.0001) and the first-pass success rate improved (64.8% vs. 43.9%, *p* < 0.0001). The time to access was also shorter in the US group (88 ± 78 s vs. 108 ± 112 s, *p* = 0.006). Hence, the RAUST trial, confirmed that US guidance improves the success and efficiency of radial artery cannulation in patients presenting for transradial catheterization (Fig. 2, Ref. [13]).

A successive meta-analysis of 12 RCTs comparing US-guided with palpation-guided radial access, which is most commonly performed for hemodynamic monitoring during surgery in 2432 adult participants, showed a significantly higher first-attempt success rate (risk ratio [RR] 1.35, 95% confidence interval [CI] 1.16–1.57) and decreased failure rate (RR 0.52, 95% CI 0.32–0.87) using US [14].

More recently, in an observational study on patients undergoing a percutaneous coronary procedure through DRA, the access success rate was significantly higher with US guidance compared to conventional puncture (97% vs. 87%, *p* = 0.038) with no significant differences in puncture time (Fig. 2, Ref. [15]). The learning curve for DRA varies depending on the technique employed. For blind puncture, achieving a consistently high success rate of over 94% required performing at least 200 cases, underscoring the importance of cumulative practice and skill acquisition in mastering this approach [16]. In contrast, when using ultrasound-guided DRA, a threshold of just 50 cases was sufficient for already skilled radial operators to establish a reliable and consistent procedural method [17]

## 4. Obtaining DRA

### 4.1 Patient Selection 

**Fig. 3.**
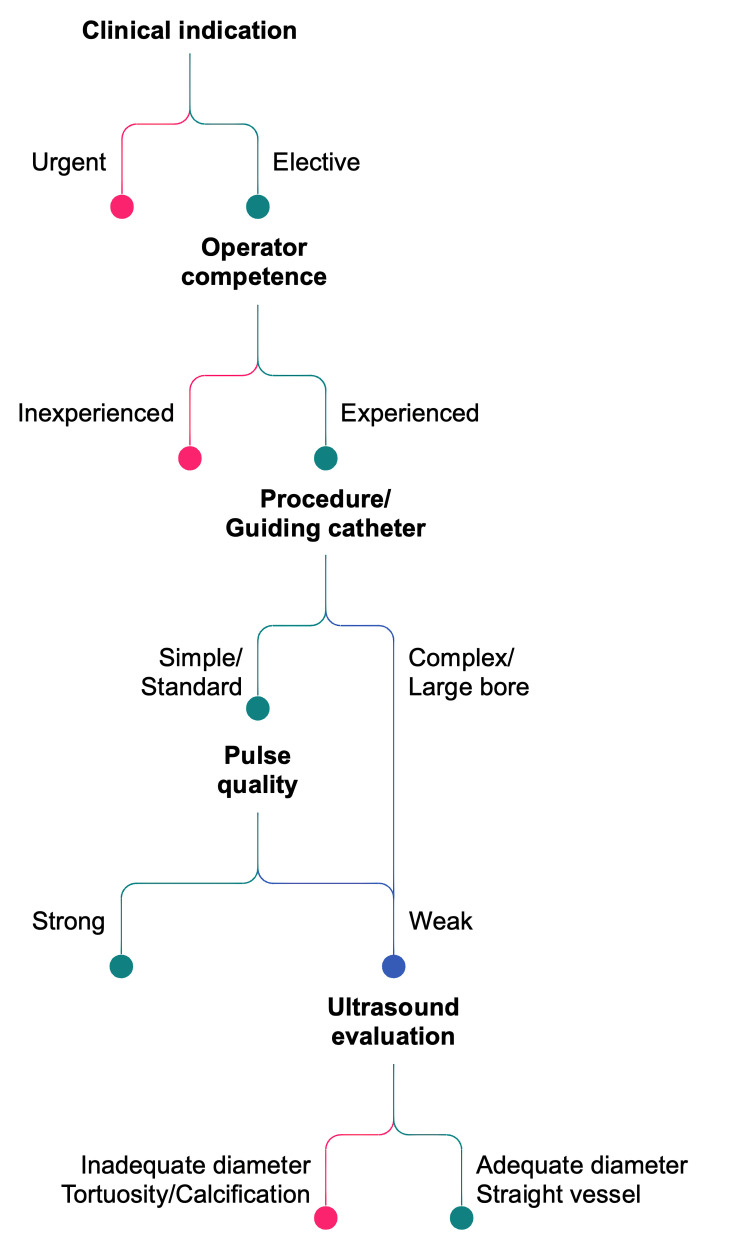
**Patient selection flow chart**. This flow chart provides a structured approach to patient selection, outlining key decision-making steps to optimize procedural outcomes based on clinical and anatomical criteria.

DRA can serve as the primary vascular access for most coronary and peripheral procedures. The decision to select DRA should follow a structured process, summarized in Fig. 3, which outlines the key steps guiding operator choice according to patient presentation, arterial anatomy, and procedural requirements.

As illustrated in Fig. 3, the first consideration is the clinical context,whether the procedure is elective, urgent, or performed in an emergency setting. In acute situations, the operator may favor the most familiar and time-efficient access, whereas in stable or elective cases, DRA can be prioritized to maximize long-term radial artery preservation.

The second decision point addresses the procedural complexity. While diagnostic and routine PCI can be accomplished with 5–6 Fr systems, complex interventions may require larger guiding catheters, which remain feasible through the distal radial artery when the vessel caliber is sufficient.

Finally, anatomical evaluation plays a central role in determining feasibility. Pulse palpation at the anatomical snuffbox offers a rapid bedside assessment and remains the simplest first step. Ultrasound can subsequently refine the evaluation by confirming vessel diameter, visualizing its course, and identifying assess tortuosity or calcification before proceeding with puncture. A distal radial artery measuring at least 1.5 mm in diameter generally provides a practical threshold for comfortable sheath insertion. While not based on a strict evidence-based cutoff, this value reflects operator experience and approximates the external diameter of a thin-walled 4 Fr introducer such as Prelude Ideal (Merit Medical) or Rain (Cordis), the smallest radial sheaths currently available in interventional cardiology. Arteries smaller than this may not necessarily preclude access but can predispose to spasm, pain, or technical difficulty.

This structured approach, as depicted in Fig. 3, ensures that DRA selection is based on an objective assessment of both patient and procedural characteristics rather than operator preference alone. It integrates clinical urgency, procedural complexity, and arterial anatomy into a single, reproducible framework that supports consistent, safe access planning.

### 4.2 Ultrasound Evaluation 

As already mentioned, US guidance provides key benefits for achieving successful DRA. It enables precise identification of the puncture site, accurate measurement of vessel size, and detection of anatomical features, such as tortuosity or medial calcification, that may increase complication risk. US also allows real-time visualization of the artery and its surrounding structures, helping the operator anticipate challenges and plan a controlled puncture.

Because the distal radial artery lies superficially, an 8–11 MHz spectral Doppler with a depth setting of 2–4 cm is typically sufficient for most cases. A linear transducer is generally preferred due to the vessel’s proximity to the skin.

Several portable US options are available, including wireless systems, transducers that connect to tablets, and handheld wireless devices compatible with multiple platforms.

The setup should be ergonomic and seamlessly integrated into the workflow, allowing the operator to fully realize the benefits of US guidance without unnecessary delays.

**Fig. 4.**
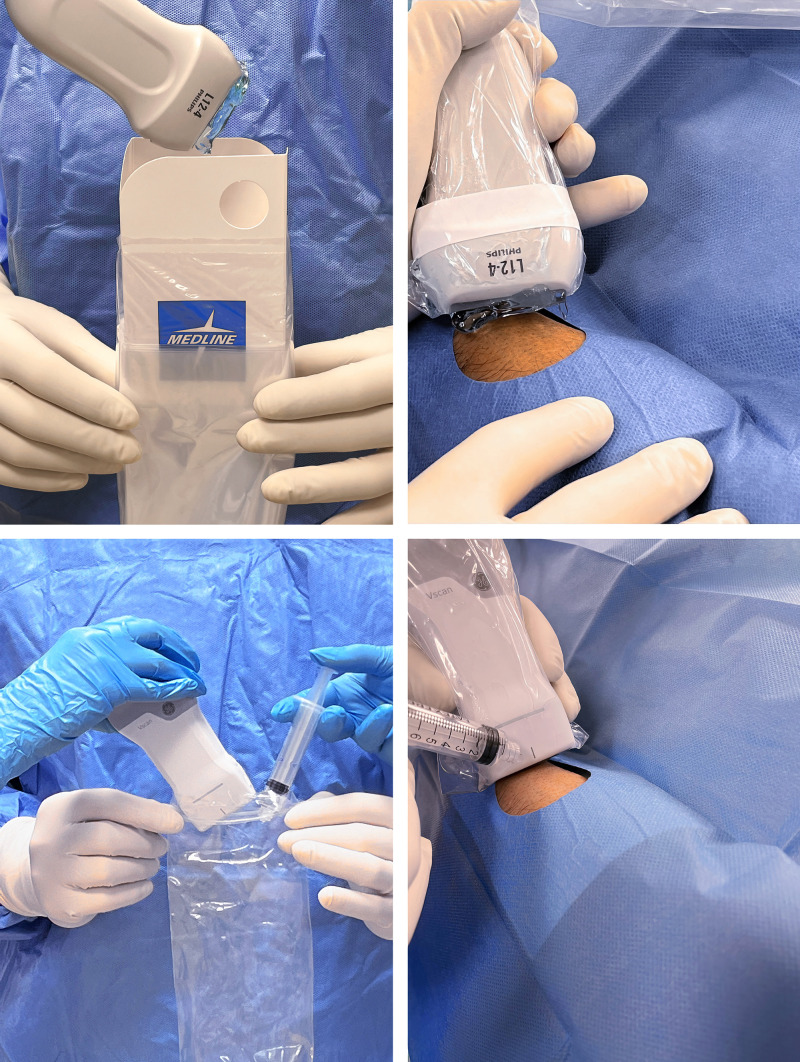
**Preparation and use of the ultrasound transducer for distal radial artery puncture**. To ensure optimal sound wave transmission and clear imaging, gel is applied to the transducer before it is inserted into a sterile sheath (upper left panel) and again before it is laid on the skin (upper right panel). Alternatively, one milliliter of sterile saline can be poured into a sterile bag before inserting the transducer (lower left panel) and a few milliliters can be poured onto the skin prior to transducer positioning (lower right panel). This alternative method provides comparable image quality while reducing slippage on the convex dorsum of the hand.

**Fig. 5.**
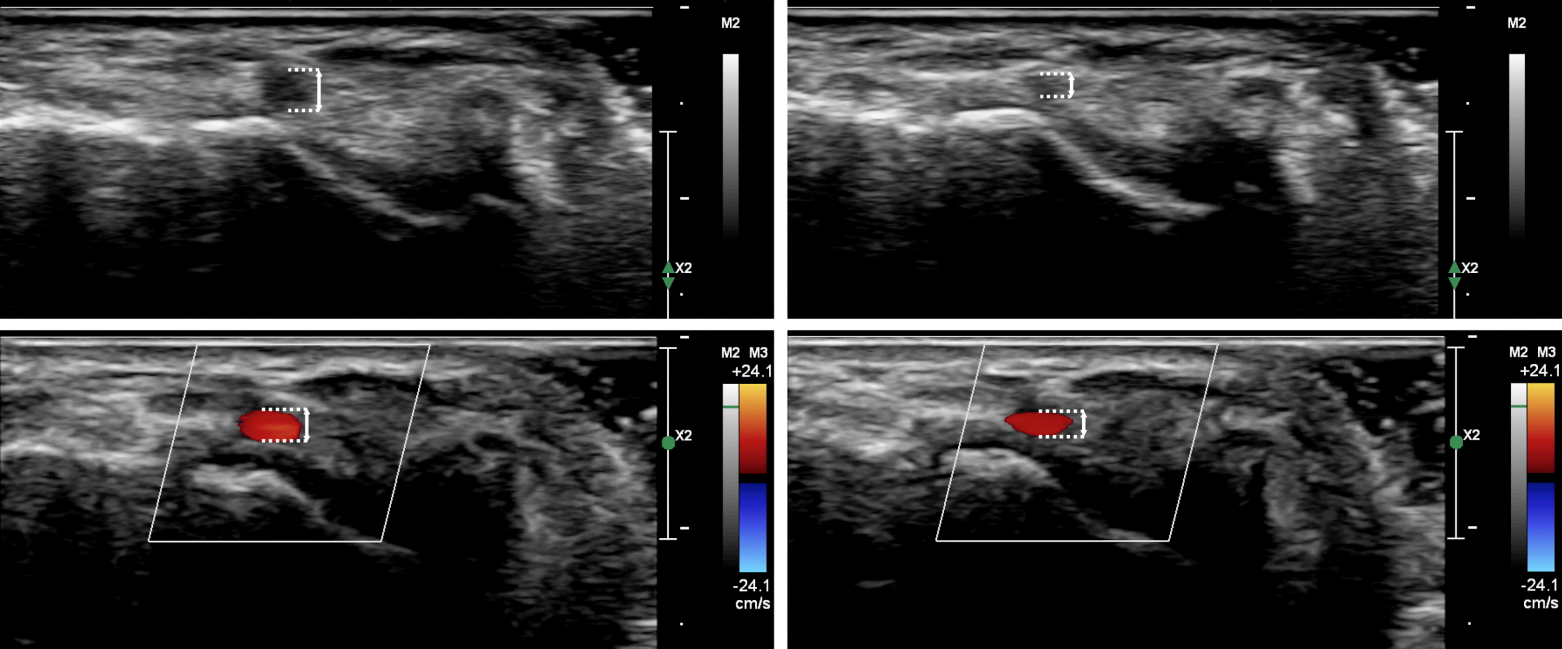
**Effect of transducer pressure on ultrasound imaging of the distal radial artery**. The left panels show grayscale (upper left) and color mode (lower left) ultrasound images of the distal radial artery in the anatomical snuffbox with the transducer lightly placed on the skin. The right panels show grayscale (upper right) and color mode (lower right) images of the same artery under some transducer pressure, resulting in moderate arterial compression and reduction in diameter. Unlike echocardiography, where a certain transducer pressure is often applied, minimal pressure is recommended for distal radial artery imaging to maintain accurate visualization and arterial patency.

**Fig. 6.**
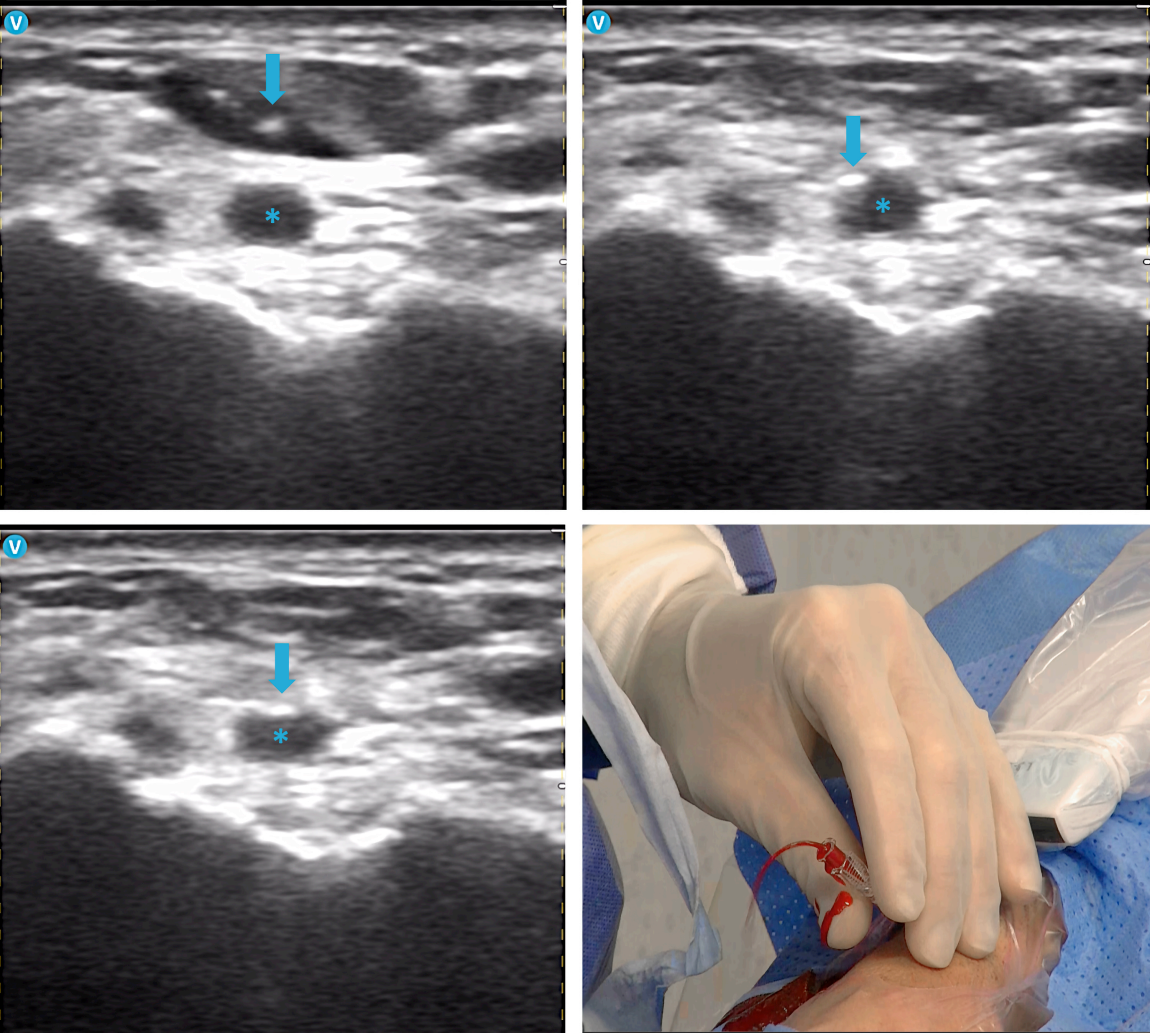
**Ultrasound-guided distal radial access**. In a transverse view, when the transducer’s centerline marker is aligned with the radial artery cross-section, the needle is advanced toward the artery (blue arrow). Ultrasound imaging allows identification of needle misalignment (upper right panel) and facilitates adjustment of the needle trajectory for optimal engagement of the arterial wall and precise entry at the 12 o’clock position (lower left panel). This approach ensures excellent blood backflow, confirming needle placement within the arterial lumen (blue asterisks).

Before starting the scan, applying saline (which is the fastest option) or conventional gel is essential to eliminate air between the probe and skin (Fig. 4). Then, the operator should first identify the borders of the anatomical snuffbox and ideally try to locate and palpate the area of maximum intensity of the distal radial artery pulse. The transducer should be held lightly against the skin, perpendicular to the imaging plane, to ensure optimal contact, which is critical. The presence of the pollicis extensor tendons and the distal radius can sometimes make it difficult to position the transducer correctly, especially in patients with very thin wrists or other unique anatomical features. Proper pressure is essential; applying too much pressure may compress the vessel and distort the surrounding structures, making puncture more difficult (Fig. 5). Transducer orientation should be confirmed by ensuring that its indicator is pointed proximally toward the snuffbox, or by applying gentle tactile pressure to the end of the transducer (Fig. 6).

Once scanning begins, depth and gain are adjusted for image quality. The operator should begin by identifying the distal radial artery in both the short and long axes. Then, assessing the vessel diameter is critical in determining the likelihood of a successful, fast, and painless puncture and cannulation.

In the initial learning phase, the operator may need to thoroughly scan the entire snuffbox area, carefully identifying all structures. However, with experience, this process becomes much faster. Before attempting vessel puncture, it is also important to evaluate the anatomical relationship of the artery with the scaphoid bone and select a puncture site where the artery is as close to the bony floor as possible to facilitate safe and efficient hemostasis afterward.

In addition, the operator should take into account the depth of the radial artery inside the snuffbox, while also identifying the presence of other anatomical structures situated between the skin and the artery that could be harmed by the needle during the procedure.

For puncture and cannulation, the administration of local anesthesia should be as close as possible to the adventitia of the distal radial artery, ideally performed under US guidance. The use of at least 1.5 mL of anesthetic helps to ensure a more comfortable procedure for the patient.

The distal radial artery can be punctured using the conventional Seldinger technique, with a 20 G puncture needle under short-axis US guidance. The operator holds the US transducer, which has initially been placed in a sterile sheath, while using the dominant hand to maneuver the 20 G needle. A stable US image with the distal radial artery clearly identified in the short-axis view, centered on the screen, is essential (Fig. 6). Ideally, the needle should be inserted approximately 1 cm distally from the transducer. However, this can be challenging in some patients, as the needle may be too close to the first metacarpal bone or the short extensor of the pollicis. To facilitate the puncture, the patient’s hand should be positioned correctly; the arm should be pronated, and the patient should be asked to gently abduct the thumb to help externalize the artery.

**Fig. 7.**
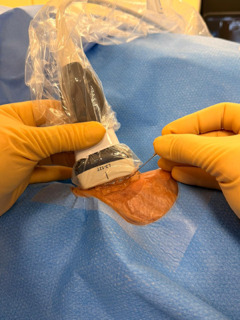
**The “ski lift” technique**. Photograph showing the transducer slightly lifted above the skin surface to allow the needle to advance beneath it.

The “ski lift” technique [18], in which the probe is lifted slightly to introduce the needle underneath, can facilitate the puncture, albeit with some trade-off in image quality (Fig. 7).

To achieve optimal angulation between the skin entry point and the artery, minimizing mechanical tension between the sheath and the arterial wall, the needle should ideally be advanced at a 30–45° angle to the skin and guided under US until it reaches its target. In practice, however, this can be difficult due to the limited surface area of the anatomical snuffbox and the surrounding strong structures. These factors often force the operator to puncture at a steeper angle, sometimes exceeding 60°.

In some cases, the needle tip may be difficult to visualize, so it may be helpful to observe the movements of the surrounding tissue by slightly tilting the needle or the ultrasound probe. Detecting the tenting of the anterior wall of the DRA is the final US step to be observed before puncturing the artery. If necessary, the transducer can be tilted along its long axis (heel-toe movement) or short axis (toggle movement) to help locate the needle tip. Successful arterial puncture is indicated by the appearance of color Doppler aliasing and a characteristic tactile sensation of release, with backflow of blood through the needle hub confirming intraluminal entry.

Occasionally, the needle may not appear in the superior mid-part of the DRA, even if the artery shows tenting, indicating that the puncture is not yet accurate. This could be due to the needle not aligning perfectly with the puncture site. Adjusting the angle of the needle can correct its position and help avoid contact with other structures or the periosteum, which could cause acute pain.

In conventional transradial access, both single- and double-wall techniques are commonly used. However, for DRA, the single-wall technique is preferred, as it reduces the risk of hematoma formation and minimizes the discomfort associated with penetrating the periosteum within the anatomical snuffbox.

After a successful puncture, a 0.021-inch plastic mini guidewire is inserted. In some cases, the operator may encounter resistance; therefore, it is critical to avoid pushing the guidewire further, as this may cause dissection or occlusive spasm of the artery. One possible solution is to adjust and decrease the needle angulation without retracting it, using gentle movements.

### 4.3 Distal Radial Ultrasound Puncture Guided Catheterization Learning Curve 

Operators may experience a learning curve when first incorporating US guidance into their practice, but this should be viewed as a valuable opportunity for growth rather than a deterrent. With time and dedication to US training, the technical learning curve can be reasonably short allowing for the systematic incorporation of a US-guided vascular approach for all types of vascular access. In a learning curve study of the DRA without ultrasound guidance, approximately 200 cases were required to achieve and maintain a consistently high success rate (>94.0%) [16]. However, with ultrasound guidance, the learning curve showed significant acceleration, with a DRA-naïve operator achieving efficacy rates comparable to the most experienced operator after just 15 cases [17]. This suggests a potential impact of ultrasound in reducing procedural variability, improving early success rates, and expediting proficiency in DRA, ultimately enhancing both training efficiency and patient outcomes.

## 5. Limitations 

Despite its clear advantages, the US guidance for DRA can present several technical and logistical challenges for operators.

From a logistical standpoint, the US device is not integrated into the main angiography system but functions as an external unit. Being an external device therefore the availability issue needs to be overcome. This separation can limit immediate availability and requires coordination to ensure the system is accessible during procedures. Another issue is that the standard echocardiography machine is typically equipped only with a cardiac probe, whereas DRA requires a dedicated peripheral vascular probe, which may not always be available in every catheterization laboratory.

Concerns are also often raised regarding procedural time. US guidance may be perceived as an additional step that increases the team’s workload, particularly for nurses responsible for preparing the device. However, once properly integrated into the workflow, US guidance actually reduces total access time by providing the operator with real-time information that facilitates rapid and accurate puncture.

From a technical perspective, US guidance can be challenging when the wrist anatomy does not allow a stable or flat probe position. In such cases, bony irregularities may degrade image quality and hinder visualization of the distal radial artery. Moreover, even with an optimal image, the transducer may occasionally obstruct the ideal puncture zone, forcing the operator to puncture very close to the probe and adopt a steeper, less ergonomic trajectory toward the vessel.

Overall, these limitations can be effectively mitigated with minimal experience and dedicated training. As operators become more familiar with the technique, both image acquisition and workflow integration improve, allowing US guidance to deliver its full procedural benefit.

## 6. Conclusion

This article reviews the rationale and methodology for implementing an ultrasound-guided approach to DRA. By enabling accurate vessel assessment before and during puncture, US guidance helps operators identify the most suitable candidates for DRA, potentially improving puncture success and procedural efficiency. Establishing US guidance as a routine component of DRA could standardize the technique, increase its overall success rate, and strengthen its clinical adoption.

Further studies are warranted to evaluate the clinical impact of a systematic US-guided vascular access strategy for the distal radial artery.
